# Erythropoietin enhances the effects of transplanted mesenchymal stem cells in an experimental model of endotoxemia

**DOI:** 10.1186/cc11707

**Published:** 2012-11-14

**Authors:** A Sorokina, A Averyanov, F Zabozlaev, A Konoplyannikov, D Akulshin, O Kuzovlev

**Affiliations:** 1 Federal Research Clinical Center FMBA of Russia, Moscow, Russia; 2 Radiologic Research Center, Obninsk, Russia

## Background

Mesenchymal stem cells (MSCs) are able to reduce systemic inflammatory response in an experimental sepsis. One of the limiting factors of MSC therapy is a high degree of apoptosis of transplanted cells. On the basis of recently detected receptors for erythropoietin (EPO) on the surface of MSCs we hypothesized that introduction of EPO together with MSCs may increase survival of grafted MSCs and improve the clinical efficacy of cell transplantation.

## Methods

Fifty Wistar male rats were randomized into five groups with 10 animals in each: Group 1 were healthy controls, Groups 2 to 5 were intraperitoneally introduced bacterial LPS 20 mg/kg. Two hours later LPS injection animals received the following intravenous treatment: 4 × 10^5 ^allogeneic MSCs (Group 3), 8.5 μg recombinant EPO-β (Group 4), MSCs and EPO in the same doses (Group 5). Surviving animals were euthanized on the fourth day. The morphological study, white blood cell count (WBC) and serum level of IL-1β, IL-2, IL-6, and TNFα measurement were performed.

## Results

**Figure 1:**
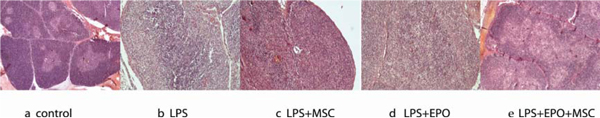
**Representative hematoxylin and eosin staining of thymus tissue 96 hours post LPS injection (×100)**.

The highest WBC was found in the group of combined treatment EPO + MSCs. The serum IL-1β level in Groups 2 and 4 was significantly higher than in healthy and treated with MSCs and EPO + MSCs animals. The main results of the morphologic analysis of different tissues in the groups are presented in Table 1. The most significant differences in the LPS + EPO group were found in the lymphoid tissue - considerable hyperplasia of spleen white pulp and thymus cortex (Figure 1).

**Table 1:** Morphometric characteristics of the examined tissues in the experimental groups

	Controls	LPS	LPS + MSCs	LPS + EPO	LPS + EPO +MSCs
Alveolar septal thickening (μM)	10.32 ± 2.05	31.25 ± 2.9	15.3 ± 2.29*	28.6 ± 4.1	10.82 ± 1.08**
Kidney tubular dystrophy (point)	0	2.54 ± 0.31	2.22 ± 0.29	1.90 ± 0.12*	0.74 ± 0.19**
T-lymphocyte volume in thymus cortex (%)	86.24 ± 2.88	34.59 ± 1.34	49.37 ± 2.16	38.21 ± 1.73	81.48 ± 2.63**
Spleen white pulp volume (%)	23.95 ± 2.8	11.15 ± 3.8	19.9 ± 2.92	22.8 ± 0.98	27.15 ± 2.89*

*P < 0.05, **P < 0.01 compared with LPS group.

## Conclusion

Combined treatment with EPO and MSCs can reduce acute lung injury and kidney damage, cause hyperplasia of lymphoid tissue and enhance the immune response more than separate treatment in an experimental model of endotoxemia in rats.

